# *Proteus mirabilis* Biofilm: Development and Therapeutic Strategies

**DOI:** 10.3389/fcimb.2020.00414

**Published:** 2020-08-14

**Authors:** Reham Wasfi, Samira M. Hamed, Mai A. Amer, Lamiaa Ismail Fahmy

**Affiliations:** Department of Microbiology and Immunology, Faculty of Pharmacy, October University for Modern Sciences and Arts (MSA), Giza, Egypt

**Keywords:** *Proteus mirabilis*, crystalline biofilm, CAUTI, antivirulence, quorum sensing, bacteriophage

## Abstract

*Proteus mirabilis* is a Gram negative bacterium that is a frequent cause of catheter-associated urinary tract infections (CAUTIs). Its ability to cause such infections is mostly related to the formation of biofilms on catheter surfaces. In order to form biofilms, *P. mirabilis* expresses a number of virulence factors. Such factors may include adhesion proteins, quorum sensing molecules, lipopolysaccharides, efflux pumps, and urease enzyme. A unique feature of *P. mirabilis* biofilms that build up on catheter surfaces is their crystalline nature owing to their ureolytic biomineralization. This leads to catheter encrustation and blockage and, in most cases, is accompanied by urine retention and ascending UTIs. Bacteria embedded in crystalline biofilms become highly resistant to conventional antimicrobials as well as the immune system. Being refractory to antimicrobial treatment, alternative approaches for eradicating *P. mirabilis* biofilms have been sought by many studies. The current review focuses on the mechanism by which *P. mirabilis* biofilms are formed, and a state of the art update on preventing biofilm formation and reduction of mature biofilms. These treatment approaches include natural, and synthetic compounds targeting virulence factors and quorum sensing, beside other strategies that include carrier-mediated diffusion of antimicrobials into biofilm matrix. Bacteriophage therapy has also shown successful results *in vitro* for combating *P. mirabilis* biofilms either merely through their lytic effect or by acting as facilitators for antimicrobials diffusion.

## Introduction

The genus *Proteus* encompasses rod-shaped Gram-negative bacteria that belong to the family *Enterobacteriaceae* (Penner, 2005). They are widely spread in the environment mainly in water, soil and the gastrointestinal tracts of humans and animals (Drzewiecka, 2016). Of the human gut microbiota, *Proteus* species comprise <0.05% in healthy subjects (Yatsunenko et al., 2012). Among all *Proteus* species, *Proteus mirabilis* is the most frequent cause of human infections (Jacobsen and Shirtliff, 2011). It is an opportunistic pathogen that is implicated in various human diseases of the respiratory tract, gastrointestinal tract, eye, ear, and skin among others (O'hara et al., 2000).

*P. mirabilis* is also a common cause of complicated urinary tract infections (UTIs) in patients with anatomical or functional problems (Jamil et al., 2020). This is particularly problematic in patients undergoing long-term indwelling urinary catheterization who may develop catheter-associated urinary tract infections (CAUTIs). Such infections are complicated by the unique ability of *P. mirabilis* to form crystalline biofilms eventually leading to encrusted and blocked catheters (Jones et al., 2007). Patients may then suffer from urine retention and reflux that are accompanied by painful distension of the bladder and pyelonephritis. Fatal complications may then be imminent such as septicemia and endotoxic shock (Chen et al., 2012). Moreover, it may cause trauma to the urethra and bladder mucosa upon removal of the catheter (Vaidyanathan et al., 2010). More nursing visits and emergency referrals may also be demanded for replacement of blocked catheters. In addition, crystalline biofilms have been linked to the persistence of *P. mirabilis* in the urinary tract through protection from antibiotics and the host immune response (Jacobsen et al., 2008).

Intestinal colonization by *P. mirabilis* provides a reservoir for intermittent colonization of the periurethral region. During insertion, urinary catheters are contaminated by the organism that is subsequently transmitted to the urinary bladder (Mathur et al., 2005). Adhesion to catheter surface or bladder epithelium induces exopolysaccharides production and biofilm formation (Nicolle, 2014).

Biofilms are well-organized structures of single- or multi-species microbial communities in which microbial cells are irreversibly attached to a substratum and to each other. Within biofilms, cells are embedded in a self-produced matrix of extracellular polymeric substances including: polysaccharides, proteins, lipids, and extracellular DNA (Flemming and Wingender, 2010). Formation of biofilms is a multistage process that starts by reversible bacterial adhesion to biotic or abiotic surfaces followed by an irreversible attachment, formation of microcolonies and finally development of a mature biofilm. The surface of the mature biofilm then starts to shed free bacteria to disseminate into other favorable environmental conditions (O'toole et al., 2000).

Biofilm formation is employed by some microbial species for surviving harsh environmental conditions and enhancing resistance to antibiotics as well as the host immune system. Antimicrobial resistance of biofilm-associated microorganisms has been found to be 10–1,000 times higher than their planktonic counterparts (Hoiby et al., 2010). The reasons behind this dramatic increase in resistance may be related to the biofilm matrix that hinders the penetration of antimicrobial agents through biofilm layers and the physiological attributes of microbial cells within biofilms especially persister cells (Tseng et al., 2013).

## Biofilm Formation

**Figure 1:**
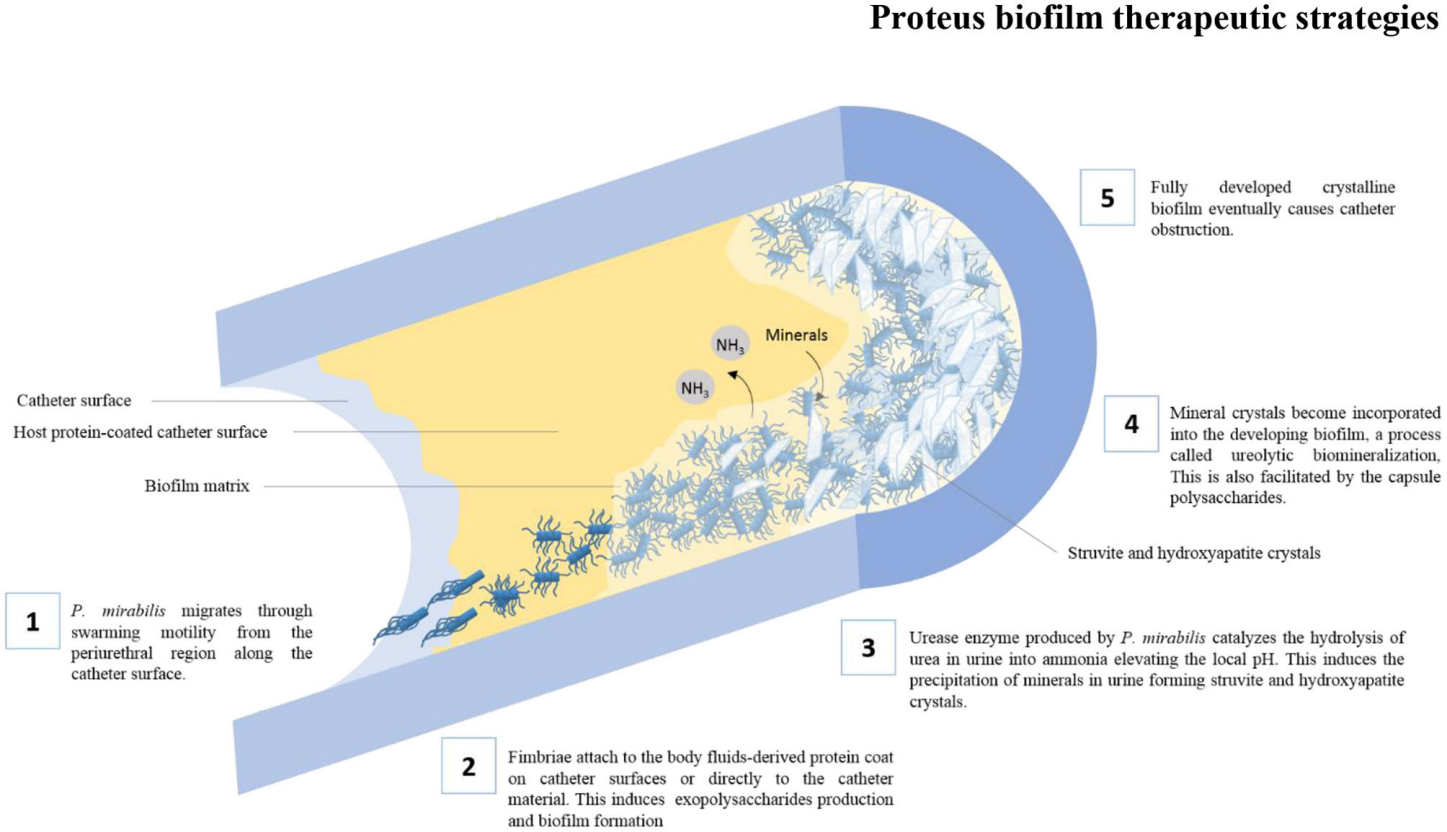
The role of various virulence factors in the formation of crystalline biofilms by *P. mirabilis* on catheter surfaces.

Among the huge arsenal of virulence factors employed by *P. mirabilis* to cause Catheter Associated Urinary Tract Infections (CAUTIs), some virulence factors have been linked to their ability to form biofilms, such as swarming motility, fimbriae, urease production, capsule polysaccharide, and efflux pumps. The role of various virulence factors in *P. mirabilis* biofilm formation is illustrated in Figure 1.

*P. mirabilis* is particularly well known for its distinctive swarming ability. Growth of the organism on solid surfaces triggers the differentiation of the short rod-shaped “swimmer cells” into highly elongated and hyper-flagellated “swarmer cells” that are capable of aligning themselves in multicellular rafts. These rafts of cells can migrate rapidly over solid surfaces in a coordinated manner (Stickler and Hughes, 1999; Harshey, 2003) The swarming motility may, therefore, facilitate the migration of *P. mirabilis* from the periurethral region along the catheter surface into the urinary bladder initiating CAUTIs (Jones et al., 2005). Loss of the swarming ability due to mutations have been linked to the failure of *P. mirabilis* to migrate over catheter surfaces (Jones et al., 2004). Moreover, swarmer cells often show higher expression of virulence factors that further enhances their ability to adhere to catheter surfaces and bladder epithelium (Allison et al., 1992; Fraser et al., 2002). However, some studies have found that swarming must be repressed in order for *P. mirabilis* to remain attached to catheter surfaces and initiate biofilm formation (Liaw et al., 2003; Jones et al., 2005).

The initial stage in the formation of biofilms on catheter surfaces is the attachment of fimbriae (adhesins) to the body fluids-derived protein coat on catheter surfaces (Donlan, 2002) or directly to the catheter material (Downer et al., 2003). At least 17 fimbrial operons were identified in the genome of a *P. mirabilis* isolate by whole genome sequencing. This is mostly the highest among all sequenced bacterial genomes (Pearson et al., 2008; Scavone et al., 2016) and is clearly reflected by its greatest ability to adhere to catheter surfaces among Gram-negative bacteria (Roberts et al., 1990). The most extensively studied fimbriae are mannose-resistant/Proteus-like (MR/P) fimbriae, mannose-resistant/Klebsiella-like (MR/K) hemagglutinins (Old and Adegbola, 1982), *P. mirabilis* fimbriae (PMF) (Bahrani et al., 1993), uroepithelial cell adhesins (UCA)/nonagglutinating fimbriae (NAF) (Wray et al., 1986), and ambient-temperature fimbriae (ATF) (Massad et al., 1996).

Expression of MR/K hemagglutinins have been linked to the attachment of *P. mirabilis* to catheter surfaces to initiate biofilm formation during CAUTIs (Jacobsen et al., 2008). However, whether the MR/K hemagglutination pattern is mediated by a specific type of fimbriae or more than one fimbriae type is still unknown. The exact nature of the gene(s) related to the MR/K hemagglutination pattern is also yet to be identified (Schaffer and Pearson, 2015). Both MR/P fimbriae and PMF have been found to play an important role in the selective adherence of *P. mirabilis* to the bladder epithelium (Sareneva et al., 1990; Rozalski et al., 1997). In a study by Jansen et al. (2004), MR/P fimbriae have been found to be neither necessary nor sufficient to initiate biofilm formation. The same study has yet concluded that their constitutive expression enhanced biofilm formation. The crucial role of MR/P in biofilm formation by *P. mirabilis* was evidenced by the smaller biofilms formed by MR/P mutants as compared to the wild type strains (Scavone et al., 2016). In contrast, the same study has reported enhanced biofilm formation in isogenic mutants unable to express PMF. UCAs, also named nonagglutinating fimbriae (NAF) (Tolson et al., 1995), were found to be structurally homologous to intestinal colonization fimbriae of *E. coli* (Cook et al., 1995). Therefore, it has been suggested that they facilitate the intestinal colonization by *P. mirabilis* thus forming a reservoir of organisms that can potentially cause CAUTIs (Coker et al., 2000). More recently, Wurpel et al. (2016) identified a new UCA-like fimbriae (UCL) in uropathogenic *E. coli* (UPEC). The authors demonstrated their ability to promote biofilm formation on abiotic surfaces and their involvement in the attachment of UPEC to uroepithelial cells. Meanwhile, the ability to form biofilms was found to be lower in UCA mutant *P. mirabilis* grown in artificial urine compared to wild type strains (Scavone et al., 2016). Despite their initial report that ATF have no role in *P. mirabilis* UTIs (Zunino et al., 2000), Scavone et al. (2016) have later demonstrated their role in adhesion and biofilm formation on abiotic surfaces. This could rather be linked to their possible role in the survival of *P. mirabilis* in the environment as suggested by their optimal expression temperature (23°C) (Rocha et al., 2007).

A characteristic feature of *P. mirabilis* is its unique ability to form unusual crystalline biofilms that usually lead to encrustation and obstruction of catheters thus making CAUTIs more complicated. Two virulence factors known to be role players in the formation of *P. mirabilis* crystalline biofilms are urease enzyme and capsule polysaccharides (CPSs) (Jacobsen and Shirtliff, 2011). Virtually all clinical strains of *P. mirabilis* produce an exceptionally potent urease enzyme. The enzyme catalyzes the hydrolysis of urea in urine into ammonia elevating the local urinary pH (Rozalski et al., 1997). This is usually coupled with local supersaturation and precipitation of minerals normally found in urine forming struvite crystals (ammonium magnesium phosphate) and hydroxyapatite crystals (calcium phosphate) (Bichler et al., 2002). Such crystals become incorporated into the developing biofilm, a process called ureolytic biomineralization (Jacobsen and Shirtliff, 2011). In addition to its major role in crystalline biofilm formation, the strongly alkaline ammonia produced by the urease enzyme is directly toxic to mammalian cells and may cause tissue damage (Burne and Chen, 2000).

Besides the ureolytic activity of *P. mirabilis*, their CPSs have been found to accelerate mineral crystals growth (Jacobsen and Shirtliff, 2011). Owing to the acidic nature of bacterial CPSs, they have a great affinity for binding metal cations in urine via electrostatic interaction. Nevertheless, bacterial species, as *E. coli*, with high affinity for Mg^2+^, were found to inhibit struvite crystal formation at neutral pH. This is possibly due to the strong chelation of Mg^2+^ ions that become no longer available for crystal growth. In contrast, CPSs of *P. mirabilis* have lower affinity for Mg^2+^ ions. The ions are thus weakly bound to the organism. Hence, they become concentrated, yet readily released for struvite crystal formation (Dumanski et al., 1994).

Efflux systems were recently found to play a major role in *P. mirabilis* crystalline biofilms whose formation was reduced by disruption of the *bcr* gene (Holling et al., 2014). This gene encodes an MFS efflux pump known as bicyclomycin resistance protein. In *E. coli*, this efflux pump is involved in resistance to sulphonamide and bicyclomycin (Bentley et al., 1993) in addition to exporting short peptides (Hayashi et al., 2010). Being a component of the polymeric matrix produced by biofilms (Flemming and Wingender, 2010), peptide efflux may contribute to biofilm formation (Alav et al., 2018). Peptides may also be part of the quorum sensing (QS) system of *P. mirabilis* hence, their efflux may affect signaling during biofilm formation (Alav et al., 2018).

Despite the existence of some phenotypes that require intercellular coordination such as swarming, monospecies and multispecies biofilm formation, a fully defined QS system including an autoinducer signal producer and its corresponding receptor has not yet been identified in *P. mirabilis* (Schaffer and Pearson, 2015). Further research is hence required to fill this gap of knowledge. Unlike *Serratia marcescenes* where swarming motility is regulated by a homolog of the acylated homoserine lactone (AHL) based LuxI/LuxR QS system (Sakuraoka et al., 2019), homologs of this system were not identified in the genome of *P. mirabilis* (Armbruster and Mobley, 2012). However, exposure of *P. mirabilis* to N-butanoyl homoserine lactone (BHL) was found to significantly influence biofilm formation by *P. mirabilis* O18 (Stankowska et al., 2012). Another QS system widely distributed in Gram negative and Gram positive bacteria is the LuxS/LuxP or LsR system. *P. mirabilis* was found to possess the *LuxS* gene which produces the LuxS-dependent QS molecule (AI2) during swarming. However, the null mutation of this gene did not affect swarming motility or virulence (Schneider et al., 2002). On the other hand, neither *LuxP* nor *Lsr* homologs were identified in the genome of *P. mirabilis* (Pearson et al., 2008). Absence of homologs for known AI2 binding sites might indicate the possibility of utilizing this system in interspecies signaling (Pereira et al., 2013). A homolog of LuxQ, a hybrid sensor kinase, in the LuxS/LuxP QS system in *Vibrio harveyi* was also found to be encoded by *RsbA* gene in *P. mirabilis*. This gene was found to have a role in swarming and to be sensitive to bacterial density (Belas et al., 1998).

## Therapeutic Strategies

### Antimicrobial Agents

As for other biofilm-forming bacteria, biofilm formation by *P. mirabilis* is accompanied by greater resistance to antimicrobial agents. This was demonstrated by some studies (Aiassa et al., 2006). Hence, many attempts have been made for potentiating antimicrobial agents' activity against *P. mirabilis* biofilms. One attempt was to administer the antimicrobial agent in a cyclic dosing regimen in which the treatment course is interrupted by a holding time that allows persister cells to lose their tolerance to the antimicrobial agent (Lewis, 2001, 2005). This had been proposed to eliminate biofilms and their persister cells. However, for successful clearance of biofilms using this regimen, the holding time should strictly correlate to the time required for persister cells to regain their antimicrobial susceptibility (Lewis, 2001). This is complicated by the lack of a full understanding of the exact mechanisms by which tolerance is achieved (Azeredo and Sutherland, 2008). On the other hand, two consecutive applications of antimicrobials to *P. mirabilis* biofilm were found to reduce persister cells by one log only (Abokhalil et al., 2020). In the same study, combining ciprofloxacin with silver nitrate completely eradicated persister cells in planktonic populations of *P. mirabilis*. This could be attributed to the production of reactive oxygen species and increased permeability of bacterial cells both in active and dormant states under the effect of silver nitrate (Morones-Ramirez et al., 2013).

Studies were also conducted to test the effect of minimum inhibitory concentration (MIC) and sub-MIC levels of antibiotics on inhibiting the adherence of microbial cells to plastic surfaces and consequently inhibiting biofilm formation. Their effect on preformed biofilms was also widely studied. A study by Wasfi et al. (2012) tested the effect of sub-MIC levels of four antibiotics on microbial adherence and biofilm formation by strong biofilm forming clinical strains of *P. mirabilis*. The tested antibiotics included: ciprofloxacin, ceftriaxone, nitrofurantoin, and gentamicin. Of them, ciprofloxacin showed the highest activity. Up to 93% reduction in biofilm formation was achieved using a concentration of ciprofloxacin corresponding to 1/2MIC. By testing the effect of lower concentrations on the same strains, the inhibitory effect of ciprofloxacin on biofilm formation was dramatically reduced.

On the other hand Kwiecinska-Pirog et al. (2013a) tested the effect of sub-MIC levels of ciprofloxacin and ceftazidime on preformed biofilms. The antibiotics were used at concentrations corresponding to 1/8- 1/4-, 1/2-MIC levels and at the MIC level for each. The effect of the tested antibiotics on biofilms was estimated by a colorimetric assay based on cellular physiology in biofilms using 2,3,5-triphenyl-tetrazolium chloride (TTC). Treatment of preformed biofilm by ciprofloxacin at MIC value showed significant reduction in microbial viability. In contrast, lower concentrations (1/8 MIC) of ciprofloxacin were found to increase TTC color intensity reflecting increased viability compared to the control. However, the validity of using TTC for assessing *P. mirabilis* biofilms is questionable. Only few studies used this method for *P. mirabilis* (Kwiecinska-Pirog et al., 2013b, 2014) and none of the published studies optimized this method for this organism. In contrast, this method was optimized for biofilm assays of *Pseudomonas aeruginosa* (Sabaeifard et al., 2014) and *Campylobacter jejuni* (Brown et al., 2013) by adjusting the incubation time of biofilms with TTC to 5 and 24 h, respectively. It is noteworthy here, that only 2 h incubation time was applied by Kwiecinska-Piróg and his coworkers.

The profound inhibitory effect of sub MIC concentrations of ciprofloxacin on *P. mirabilis* biofilms in comparison to other classes of antimicrobial agents could be attributed to urease inhibition (Abdullah et al., 2016) and reduction of flagellar formation (Horii et al., 2003). An added advantage to ciprofloxacin use in the treatment of infections associated by *P. mirabilis* biofilms was reported by Irwin et al. (2013). They found that both bacteriostatic and bactericidal effects of ciprofloxacin on *P. mirabilis* biofilm forming cells were enhanced by increasing pH from 5 to 9. This was reflected by a 10-fold reduction in the minimum biofilm eradication concentration (MBEC). The enhanced activity at high pH levels makes ciprofloxacin a good candidate for use in treatment of *P. mirabilis* CAUTIs in which urease enzyme is the main player. Irwin et al. (2013) justified their findings by the acquisition of a negative net charge by ciprofloxacin molecules at high pH that enables them to readily diffuse through the biofilm matrix. However, at lower pH the antibiotic molecules acquire a positive net charge and become strongly attracted to the negatively charged exopolymeric biofilm matrix. This hinders the penetration of the antibiotic through the biofilm matrix. In contrast to the previously mentioned results, another study in artificial urine revealed that the penetration of ciprofloxacin into *P. mirabilis* biofilms was reduced at high pH due to biomineralization. This was evident among urease producing strains compared to urease non-producers by LIVE/DEAD stain (Li et al., 2016).

Several strategies have been employed for controlling biofilm formation in catheters using antimicrobial agents, among them are adsorbing antimicrobial agents on catheter surfaces and using modified coating material thus inhibiting bacterial adherence. Only silver and antibiotic coated catheters have been tested clinically (Singha et al., 2016). Filling catheter balloons with water containing soluble antimicrobials was found to allow adhesion of the antimicrobial agent to the catheter surface thus preventing subsequent adhesion of microorganisms (Stickler et al., 2003; Williams and Stickler, 2008). An example of this strategy is using a mandelic/lactic acid mixture in silicone catheters which gave debatable results (Robertson and Norton, 1990; Stickler and Hewett, 1991). The second strategy is using modified catheter coating with antimicrobial effect to prevent *P. mirabilis* colonization. Silver coatings and nitrofurazone-coated silicone catheters have shown significant prevention of catheter-associated bacteriuria during the short term catheterization of hospitalized patients (Stensballe et al., 2007; Schumm and Lam, 2008; Johnson et al., 2012). However, crystalline biofilms could still be formed on hydrogel/silver coated Foley catheters, particularly when the antimicrobial coating fails to diffuse out of the catheter material into the catheter lumen (Morgan et al., 2009). On the other hand, incorporating ciprofloxacin in biodegradable waterborne polyurethane was found to enhance the inhibitory effect of ciprofloxacin on biofilm formation by *P. mirabilis* in artificial urine medium *in vitro* (Xu et al., 2019).

### Phytochemicals

Historically, plants have been considered as a rich source of a wide range of bioactive compounds, known as phytochemicals (Ahmed and Urooj, 2009). Among their diverse applications, phytochemicals have particularly gained much interest for their antibiofilm activity. This activity was attributed to their ability to inhibit virulence factors including: microbial adherence to surfaces, quorum sensing, urease activity and exopolysaccharide matrix production (Table 1). Few studies reported the development of resistance to antivirulence compounds (Beceiro et al., 2013).

**Table 1:** Overview of phytochemicals assessed for *Proteus mirabilis* biofilm control.

**Phytochemical**	**Source of phytochemical**	**Possible mechanism**	**References**
Allicin	Garlic	Inhibition of urease enzyme therefore affecting biomineralized biofilm and hence inhibit biofilm formation	Ranjbar-Omid et al., 2015
A2-type Proanthocyanidins	Cranberry (*Vaccinium macrocarpon*)	Reducing adherence of bacteria to epithelial cells and reduce urease production	Nicolosi et al., 2014
		Reduce motility and increase biofilm formation on plastic surfaces	O'may et al., 2016
Phenanthrene and fatty acid	*Hyptis suaveolens*	Inhibition of motility	Salini et al., 2015
Pyrrolo(1,2-a)pyrazine-1,4-dione,hexahydro-3-(2-methylpropyl)	*Nocardiopsis* sp.	Inhibiting biofilm and antibacterial effect	Rajivgandhi et al., 2018
Curcumin	*Curcuma longa*	Reducing EPS	Packiavathy et al., 2014
Latex extract	*Euphorbia trigona*	Inhibiting biofilm formation, reduce ureasing enzyme and inhibiting swarming	Nashikkar et al., 2011
Methanolic extract of the dried fruits of Capparis spinosa	*Capparis spinosa*	Reducing EPS production	Issac Abraham et al., 2011
Linalool	Floral aromatic and spice plants	Reducing motility	Durgadevi et al., 2019
N-acetyl cysteine (NAC) and dipropyl disulphide	Onion oil	Inhibiting urease, motility, and adherence	Abdel-Baky et al., 2017
Extract	*Ibicella lutea*	Inhibiting biofilm formation and inhibit swarming	Sosa and Zunino, 2009

Being the first step in biofilm formation, inhibiting adherence to surfaces was found to reduce the overall capacity of microorganisms to form biofilms. Numerous studies have shown a correlation between the consumption of cranberries (*Vaccinum macrocarpon*) and the prevention of UTIs (Nowack and Schmitt, 2008; Tempera et al., 2010). The underlying mechanism has not yet been definitively established. Though, it has been proposed that cranberries interfere with bacterial adhesion to uroepithelial cells leading to failure of bacterial cells to colonize the urinary tract (Nicolosi et al., 2014). Such effect was correlated to two components of cranberries including fructose, that blocks type 1 fimbriae (sensitive to mannose), and the proanthocyanidins (PACs), that inhibit the mannose-resistant P-fimbriae (Ermel et al., 2012). In a study by O'may et al. (2016), cranberry treatment of *P. mirabilis* reduced adhesion to HT1376 cell line by up to 75%. Motility and urease activity were also reduced in response to the same treatment. On the other hand, cranberry treatment in the mentioned study enhanced biofilm formation by *P. mirabilis* on plastic surfaces. Such effect was attributed to a transient reduction in bacterial motility. This was also clinically evident by higher rate of *P. mirabilis-*induced catheter encrustation in urine from individuals that had consumed cranberry juice than those who consumed an equivalent amount of water (Morris et al., 1997; Morris and Stickler, 2001).

Another phytochemical compound that showed inhibitory effect on *P. mirabilis* biofilms is the extract of *Ibicella lutea*, a native plant in South America. The antibiofilm activity of *Ibicella lutea* was coupled to its ability to inhibit the swarming differentiation of *P. mirabilis* (Sosa and Zunino, 2009). As mentioned before, swarming motility is employed by the organism to move across solid surfaces until it reaches the site of colonization. This is followed by inhibition of flagellar movement and the formation of biofilms (Guttenplan and Kearns, 2013).

The alkaline urinary pH, imparted by the activity of urease enzyme, plays an essential role in the development of crystalline biofilms by *P. mirabilis*. Accordingly, inhibiting urease production and subsequent pH elevation could be an important target in preventing biofilm formation. A phytochemical compound that targets urease production in *P. mirabilis* is allicin, extracted from garlic. At sub-MIC levels, it could reduce biomineralized biofilm formation by up to 35%, as reported by Ranjbar-Omid et al. (2015). The authors have also reported complete eradication of established biofilms at higher concentrations of allicin. A unique feature of allicin, among a few other compounds, is its ability to penetrate bacterial cell membrane and inhibit urease enzyme intracellularly (Miron et al., 2000). The clinical use of allicin is, however, hampered by its instability in biological fluids (Rosen et al., 2001). Inhibition of urease production and biofilm formation in *P. mirabilis* were also evident for N-Acetyl Cysteine (NAC) and dipropyl disulfide, two components in onion oil (Abdel-Baky et al., 2017). In addition to its urease-inhibiting activity, NAC disrupts disulfide bonds in the extracellular polymeric matrix thereby disrupting biofilm architecture (Kregiel et al., 2019).

Despite the lack of clear information about a well-defined QS system in *P. mirabilis*, anti-quorum sensing effects of some phytochemical compounds have been reported by several studies. Such findings were based on their ability to inhibit violacein production by *Chromobacterium violaceum* (ATCC 12472) without affecting microbial growth. To the best of our knowledge, none of the published studies investigated AHL production in *P. mirabilis* by testing the effect of *P. mirabilis* cultures on the biosensor mutant *Chromobacterium violaceum* CV026 strain.

In a study by Salini et al. (2015), *Hyptis suaveolens*, an aromatic perennial herb found in tropical America, was evaluated for potential therapeutic value. The plant extract showed a potent anti-quorum sensing activity, using the QS biosensor *Chromobacterium violaceum* (ATCC 12472), and could decrease biofilm mass of *Proteus* sp. in a dose-depended manner. At 200 μg/ml, *Hyptis suaveolens* extract could effectively dislodge the biofilm of *P. mirabilis* and *P. vulgaris* by 42% and 59%, respectively, without affecting microbial growth.

In a study carried by Packiavathy et al. (2014), sub-MIC levels of curcumin, a potent QS inhibitor, were found to reduce polysaccharide production and swarming motility, hence reducing biofilm formation. The compound could also inhibit the formation of microcolonies and disrupt preformed *P. mirabilis* biofilms.

A phytochemical compound, Pyrrolo [1,2-a] pyrazine-1,4-dione, hexahydro-3-(2-methylpropyl), extracted from endophytic actinomycete *Nocardiosis* sp., was found to inhibit microbial adherence and biofilm formation. The research group proved the QS inhibitory effect of this compound using the QS biosensor *Chromobacterium violaceum* (ATCC 12472) (Rajivgandhi et al., 2018).

*Capparis spinose* is a spice reported to have a number of potentially useful medicinal attributes including antioxidant, antifungal, anti-inflammatory, anti-diabetic, and anti-obesity activities. Issac and coworkers have demonstrated the anti-quorum sensing activity of *C. spinose* extract. Under the effect of the extract, clear alteration in *P. mirabilis* biofilm architecture was revealed by confocal laser scanning microscopy (CLSM). This was associated by inhibition of biofilm formation and exopolysaccharide production to 70 and 67%, respectively. One of the bioactive metabolites in *C. spinose* to which the negative impact on biofilm formation was attributed is vanillic acid (Issac Abraham et al., 2011). The anti-quorum sensing activity of vanillic acid was reported by other studies as well (Choo et al., 2006; Sethupathy et al., 2017).

*Euphorbia trigona* latex has been used for the treatment of infectious and inflammatory diseases in India as part of their pharmacopeia. Inhibition of swarming motility and virulence factors such as ureases activity in *P. mirabilis* in the presence of *E. trigona* latex was reported by Nashikkar et al. (2011). Such effects were attributed to some components of the extract with anti-quorum sensing activity.

Linalool is a monoterpene alcohol found in the essential oils of many floral aromatic and spice plants (Peana et al., 2002). The inhibitory effect of linalool on *P. mirabilis* virulence factors was the subject of a recent study by Durgadevi et al. (2019). Up to 75% inhibition of crystalline biofilm formation was achieved by linalool at a concentration of 0.4 mg/mL. Transcriptome analysis showed downregulation of the genes *flhD, flhB, speA, rsbA, ureR, hpmB*, and *hpmA*. In turn, the mentioned genes affect virulence properties of *P. mirabilis* such as motility, biofilm formation, urease activity, and hemolysin production.

### Apitherapy

One of the natural alternatives to antibiotics for controlling biofilms is apitherapy, a practice in which bee products such as honey, pollen, and propolis are used for prevention or treatment of diseases (Malone and Tsai, 2016).

Honey is a natural compound whose antibiofilm activity was the subject of many studies. The inhibitory effect of several types of honey of different botanical origins on *P. mirabilis* biofilms was evaluated by Majtan and co-workers. All honey samples were able to significantly inhibit the adherence of *P. mirabilis* cells to plastic surfaces at sub-MIC value of 10% (w/v). Moreover, partial detachment of preformed biofilms was achieved at a concentration of 50% (w/v) of each. Among the tested types of honey, the most powerful anti-biofilm properties were shown by manuka honey, produced from the nectar of the manuka tree. It could completely eradicate all biofilm-embedded *P. mirabilis* cells. Methylglyoxal, the major antibacterial component of Manuka honey, is thought to readily diffuse through the established *P. mirabilis* biofilm matrix and kill bacterial cells (Majtan et al., 2014).

Another proof on the efficacy of Manuka honey in disrupting preformed biofilms of *P. mirabilis* was presented by Abbas (2014) who also reported the same effect for the Egyptian clover honey. Manuka honey was also acknowledged by another study for its immunostimulant effect and enhancing wound healing (Gannabathula et al., 2012).

Another bee-product that is known to be one of the richest sources of active compounds, such as flavonoids and phenolic compounds, is the ethanol extract of propolis (EEP) (Freires et al., 2016). Kwiecinska-Pirog and his coworkers have evaluated the effect of the EEP on the formation of *P. mirabilis* biofilms as well as its effect on preformed biofilms (Kwiecinska-Pirog et al., 2019). Reduction in biofilm formation was observed at the concertation range of 2.5–100 mg/mL, while higher concentrations (25–100 mg/mL) could successfully reduce preformed biofilms. Meanwhile, biofilm formation by *P. mirabilis* was enhanced by treatment with a low concentration (1.5 mg/mL) of EEP. This could be attributed to the inhibition of bacterial motility at lower extract concertation that enhances the adherence of cells to surfaces (De Marco et al., 2017).

### Repurposed Drugs

Drug repurposing or drug repositioning is the use of approved drugs for treatment of medical conditions other than those for which they have been originally indicated. This offers time and cost savings by skipping some phases of clinical trials required for approval of new drugs (Pushpakom et al., 2019).

Ambroxol is a mucolytic and expectorant agent commonly used in patients with asthma and chronic bronchitis. It has been reported to interfere with biofilm formation as a result of its ability to inhibit adhesion, QS and biofilm matrix production (Lu et al., 2010). In a study by Abbas (2013), ambroxol inhibited biofilm formation and eliminated pre-formed biofilms in a dose-dependent manner. Biofilm inhibition and eradication of preformed biofilm of *P. mirabilis* reached 80, and 76%, respectively.

Other repurposed drugs with potential antibiofilm activity include fluoxetine and thioridazine which are indicated for treatment of depression and psychosis, respectively. Both were found to significantly reduce the rate of *P. mirabilis* crystalline biofilm formation on catheter model, and increase the time taken for catheter blockage. A significant reduction in swimming and swarming motilities of *P. mirabilis* was also evident after treatment with fluoxetine and thioridazine (Nzakizwanayo et al., 2017). Using molecular docking techniques, it was predicted that both drugs strongly interact with the biofilm-associated efflux system *Bcr/CflA*. This efflux system was previously linked to biofilm formation and motility in *P. mirabilis* (Holling et al., 2014).

### Phage Therapy

Phage therapy (PT) arises as one of the new alternative methods for combating bacteria using a natural predator (bacteriophage) that specifically kills bacteria (Miedzybrodzki et al., 2012). Over the last two decades, more interest in the therapeutic uses of phages has been observed. This was driven by the high prevalence of multidrug-resistant bacteria together with the inadequate discovery of new antibiotics (Skurnik et al., 2007).

Phages may be more advantageous to antibiotics in treatment of bacterial infections for several reasons: (a) Narrow spectrum of activity, thus selectively affecting pathogenic bacteria without affecting normal flora. However, the strain specificity might limit their therapeutic applications and implies the use of cocktails of bacteriophages (Alves et al., 2016; Yazdi et al., 2018). Cocktails are also used to avoid the emergence of phage-resistant strains through alteration of bacterial membrane receptors, (b) The capacity of phage multiplication at the site of infection (Hanlon, 2007; Gorski et al., 2009; Kutter et al., 2010; Harper et al., 2014), (c) Retaining activity against multidrug resistant bacteria as it employs a unique mechanism of action (Sillankorva et al., 2011), (d) low cost (Stafford, 2011), (e) Discovery of new bacteriophages is much easier and less time consuming than that of antibiotics.

Bacteriophages are ubiquitous in the environment. They are also abundant in the digestive tract of humans and animals (Viertel et al., 2014). Several studies have been attempted for the isolation of bacteriophages with the potential of combating *P. mirabilis* biofilms. Such studies employed enrichment methods in which clinical strains of *P. mirabilis* were used for the isolation of bacteriophages from sewage samples (Lehman and Donlan, 2015; Nzakizwanayo et al., 2015; Melo et al., 2016) and human feces (Morozova et al., 2016). As revealed by transmission electron microscope (TEM), the bacteriophages with activity against *P. mirabilis* biofilms, belonged to the order *Caudovirales* that encompasses tailed bacteriophages. Among which, some belonged to the family *Siphoviridae* (phages with long, flexible non contractile tail) (Maszewska et al., 2018; Yazdi et al., 2018) and others belonged to the families *Podoviridae* (phages with short stubby tail) (Kaca et al., 2011; Alves et al., 2016; Melo et al., 2016) and *Myoviridae* (phages with contractile tail) (Kaca et al., 2011; Melo et al., 2016; Maszewska et al., 2018; Alves et al., 2019). All of them were of the lytic type that replicate inside the host cells eventually causing them to rupture releasing progeny viruses (Skurnik et al., 2007; Gill and Hyman, 2010).

Different mechanisms have been suggested for the antibiofilm activity of phages. Phages can transfer through water channels to reach the biofilm-embeded bacterial cells (Chan and Abedon, 2014). They locally replicate within the cells they have reached to increase in number and spread to nearby cells reducing the total number of cells forming the biofilm. In addition, phages can express or induce the expression of depolymerizing enzymes that degrade the exopolysaccharide and facilitate the spread of phages within biofilms (Latka et al., 2017). Bacteriophages can also infect reactivated persister cells preventing relapse of infections due to this reactivation (Harper et al., 2014).

Several studies, however, showed that phages could more effectively prevent the formation of *P. mirabilis* biofilm than destroying a preformed one (Carson et al., 2010; Lehman and Donlan, 2015). This was attributed to the poor penetrability of phages within older biofilm layers (Chhibber et al., 2013). Efficient bacteriophage lysis was deficient within biofilms due to the low concentration of actively proliferating cells. This is due to the low metabolic activity within the innermost parts of biofilms (Sillankorva et al., 2004; Cerca et al., 2007). Accordingly, when administered early in the bacterial colonization step, bacteriophages were demonstrated to prevent blockage of Foley catheters due to *P. mirabilis* biofilms for over 8 days (Nzakizwanayo et al., 2015; Aniejurengho, 2016). On the other hand, established or late stage biofilm related infections were more resistant to eradication by bacteriophages (Aniejurengho, 2016).

As for the use of bacteriophages for treatment of *P. mirabilis* crystalline biofilms, several factors in the urinary tract have been found to influence phage's adsorption to host cells. Such factors include the chemical environment and pH (Jonczyk et al., 2011). Cofactor cations such as CaCl_2_ and MgCl_2_, in urine, have been found to increase the infectivity of phages as a result of higher concentration of phages on bacterial cell surface and alteration in the receptors. As a result, phages access to receptors or phage nucleic acid translocation is accelerated (Jamal et al., 2015; Yazdi et al., 2018). On the other hand, the crystals formed in urine during biofilm formation were found to inhibit adsorption of phages to their target cells.

Contradictory results about the effect of pH on the activity of bacteriophages were reported by previous studies. Some of them demonstrated the inactivation of phages at extreme pH values (pH 2 and 11) and reduction of phage titer value at pH 8 by about 30%. Accordingly, the authors suggested that the effectiveness of phages may be impaired by alkaline conditions in urine during *P. mirabilis* infection. Higher *in vivo* doses of phages may then be required for biofilm eradication (Maszewska et al., 2018; Yazdi et al., 2018). On the other hand, bacteriophages isolated by Yazdi et al. (2018) showed high stability to a wide range of pH (3–11). A finding that was considered advantageous for treatment of *P. mirabilis* biofilm with fluctuation in pH. While the outer part of the biofilm is alkaline in nature, low pH is observed within the biofilm matrix due to accumulation of acidic metabolites. The stability of phages over a wide pH range then allows retention of activity during translocation through different parts of the biofilm, thus making phages good candidates for treatment of UTIs caused by *P. mirabilis* biofilms.

Two phage-based strategies were proposed by Donlan (2009) for combatting bacterial biofilm formation on catheter surfaces. The first strategy based on preventing bacterial adhesion and biofilm formation by coating the catheter surface with phages. The second one employed lytic bacteriophages as well as phage depolymerases for biofilm eradication.

So far, up to 90% reduction in biofilm formation was achieved in phage-coated *in vitro* catheter models in several studies (Carson et al., 2010; Nzakizwanayo et al., 2015; Melo et al., 2016; Thompson, 2018). A potential drawback of this strategy is the unknown and uncontrolled orientation of the phages. However, this strategy was found to eradicate early biofilm colonization and delay catheter blockage (Nzakizwanayo et al., 2015). Inconsistent findings were reported by another study carried out by Thompson (2018) who showed more adherence of bacterial cells to catheter sections pre-treated by phage, an effect that was strain dependent (Aniejurengho, 2016). A possible explanation to his finding is the formation of conditioning film of lysed bacterial cells which mask the effect of phages and provide a sticky surface for adhesion (Fernandez et al., 2017).

A more successful approach utilizing bacteriophages for fighting biofilms is the use of phage-antibiotic combinations (Yazdi et al., 2018). Failure of this approach in biofilm treatment is unlikely, as bacterial cells which show resistance to one agent will be susceptible to the other (Chhibber et al., 2013). Furthermore, while passing through biofilm layers, bacteriophages will facilitate the diffusion of the antibiotic through biofilm layers (Verma et al., 2009; Harper, 2013; Harper et al., 2014).

### Nanoparticles

Nanoscale materials (1–1000 nm) are widely used nowadays owing to their high capacity for tissue targeting (Savolainen et al., 2010). Reducing the size to nanoscale can modify the properties of materials in terms of their chemical, mechanical, electrical, structural, morphological and optical properties (Buzea et al., 2007).

Cationic dendrimeric peptides constitute a novel class of molecules with antimicrobial potential. The positive charge on the surface of such peptides is proposed to interact with negatively charged molecules on the surface of bacterial cells such as phosphate groups of Gram-negative lipopolysaccharides (Jenssen et al., 2006; Weidenmaier and Peschel, 2008). In addition, their hyperbranched nature offers chemically reactive groups to which other molecules can be attached imparting therapeutic properties (Shi et al., 2007). As antibiofilm agents, dendrimers possess several promising characteristics. Owing to their small particle size, they can readily penetrate the EPS matrix exerting some bacteriostatic activity. Dendrimers have been also found to inhibit the formation of the EPS matrix by inducing nucleic acid mutations (Limoli et al., 2014). They are also capable of inhibiting protein synthesis (Mardirossian et al., 2014). Another possible mechanism by which the cationic dendrimeric peptides may act is their detergent-like effect. They interact with the anionic components of bacterial membranes and biofilm matrix leading to loss of membrane potential. Aniejurengho (2016) has demonstrated the ability of dendrons to reduce preformed *P. mirabilis* biofilms by up to 83.5%. Thus, they provide a clinically beneficial alternative to antibiotics for eradication of preformed biofilms that is achievable only by unattainable *in vivo* concentrations (Hengzhuang et al., 2011; Belfield et al., 2015; Wu et al., 2015).

Selenium (Se) is a micronutrient metalloid. It is a structural part of several enzymes such as glutathione peroxidases, iodothyronine deiodinases, and thioredoxin reductase, which are involved in antioxidant defense, detoxification and metabolism, respectively (Messarah et al., 2012; Zhai et al., 2017). The effect of biologically synthesized (biogenic) Selenium nanoparticles (SeNPs) on *P. mirabilis* biofilms was studied by Shakibaie et al. (2015). Interestingly, it inhibited biofilm formation to 53.4%. Based on *in vitro* and *in vivo* data, biogenic SeNPs have lower toxicity than that of selenite or selenate (Shakibaie et al., 2010). As a result, biogenic SeNPs or antimicrobials loaded on the surface of SeNPs might be good candidates for use as novel antibiofilm agents.

Low concentrations of ZnO:MgO NPs have been found to affect the development of *P. mirabilis* biofilm regarding bacterial number and extracellular matrix. This has been linked to preventing the bacterium from colonizing surfaces and formation of mature biofilms. ZnO:MgO NPs were also found to affect the production of extracellular matrix, an essential component of biofilm that protects bacteria from environmental stress (Iribarnegaray et al., 2019). Reduction in bacterial numbers within biofilms may also be attributed to the antibacterial activity of the NPs. ZnO nanoparticles (ZnO NPs) have shown great antibacterial activity against *P. mirabilis* (Gunalan et al., 2012). The main mechanisms whereby ZnO NPs may exert their antibacterial activity are alteration of cell membrane integrity (Brayner et al., 2006; Irzh et al., 2010) and generation of reactive oxygen species (Sawai, 2003; Jones et al., 2008). Synergistic activity may also be achieved with MgO NPs (Pradeev Raj et al., 2018) that have also shown great antibacterial activity against both Gram-negative and Gram-positive bacteria (Hayat et al., 2018).

## Conclusion

*Proteus mirabilis* is one of the leading causes of CAUTIs. Such infections are complicated by the unique ability of *P. mirabilis* to form crystalline biofilms. Compared to their planktonic counterparts, biofilm-embedded cells are considerably recalcitrant to antimicrobial treatment and host immune response. Biofilm formation is a multistage process that starts by the reversible adhesion of bacterial cells to biotic or abiotic surfaces followed by an irreversible attachment, formation of microcolonies and finally development of a mature biofilm. Some virulence factors have been linked to the ability of *P. mirabilis* to form crystalline biofilms. Such factors include swarming motility, fimbriae, urease production, capsule polysaccharide, and efflux pumps. The inhibitory effect of different antimicrobials on *P. mirabilis* biofilms was evaluated by many studies. A broad agreement on the efficacy of ciprofloxacin in inhibiting biofilm formation and eradication of preformed biofilms was found. Showing activity at high pH levels was an added advantage. The cyclic administration of antimicrobials also showed promising results for controlling biofilms. However, the efficacy of this approach was tightly linked to better understanding of persister cells and the mechanisms underlying their tolerance to antimicrobials. Some information on the performance of persister cells in planktonic cultures of *P. mirabilis* were provided by recent studies. However, more studies are required to fill the gap of knowledge on their performance within biofilms as well as the impact of antimicrobial combinations on their viability. Other studies were concerned with the antibiofilm effect of different phytochemicals extracted from herbs used in folk medicine. Many phytochemicals showed promising antivirulence effects including effect on: motility, urease activity, production of polysaccharide and adherence. This raises the demand for conducting more studies on the effect of phytochemical-antimicrobial combinations. Such combinations might decrease the selective pressure on microorganisms upon using antimicrobials alone which leads to the development of antimicrobial resistance. Apitherapy is among the natural alternatives whose antibiofilm activity was demonstrated by some studies. Inhibition of biofilm formation and eradication of preformed biofilms of *P. mirabilis* were proven for different types of honey as well as the ethanolic extract of propolis. Repurposing of some FDA-approved drugs for controlling *P. mirabilis* biofilms was also recommended by some studies. Among these drugs are ambroxol, fluoxetine and thioridazine. Apart from their promising application in treatment of bacterial infections, the role of bacteriophages in combating biofilms was also a matter of concern. Different types of bacteriophages showing inhibitory effect on *P. mirabilis* biofilms were successfully isolated. However, their higher ability to effectively prevent the formation of *P. mirabilis* biofilm than destroying a preformed one was noted. For treatment and/or prevention of *P. mirabilis*-mediated CAUTIs, bacteriophages have shown several advantages. Most importantly are their ability to withstand high pH levels and the enhanced activity in presence of urinary cations. Effectiveness of phage-antimicrobials combinations was also evident. Finally, among the emerging fields of study in fighting *P. mirabilis* biofilms is the nanoparticles. For this purpose, the activity of different compounds with a nanoscale size was evaluated. Among which cationic dendrimeric peptides, biogenic selenium NPs and ZNO:MgO NPs showed promising results.

All therapeutic strategies mentioned in this review should be considered for controlling *P. mirabilis* biofilms. Unique mechanisms of action are employed by each allowing the use of combined strategies for better results. However, more investigations are required for examining some of these products for their *in vivo* effect and to ensure tolerability and lack of toxicity.

## Author Contributions

RW, SH, MA, and LF took part in drafting, revising, and preparation of the manuscript. The manuscript was finalized and prepared for submission by RW and SH. All authors contributed to the article and approved the submitted version.

## Conflict of Interest

The authors declare that the research was conducted in the absence of any commercial or financial relationships that could be construed as a potential conflict of interest.

## References

[B1] Abbas H. (2014). Comparative antibacterial and antibiofilm activities of Manuka honey and Egyptian clover honey. Asian J. Appl. Sci. 2, 110–115.

[B2] Abbas H. A. (2013). Ambroxol blocks swarming and swimming motilities and inhibits biofilm formation by *Proteus mirabilis* isolated from diabetic foot infection. Asian J. Pharacy Technol. 3, 109–116.

[B3] Abdel-Baky R. M. Ali M. A. Abuo-Rahma G. Abdelaziz N. (2017). Inhibition of urease enzyme production and some other virulence factors expression in *Proteus mirabilis* by N-acetyl cysteine and dipropyl disulphide. Adv. Exp. Med. Biol. 973, 99–113. 10.1007/5584_2016_197 28190143

[B4] Abdullah M. A. El-Baky R. M. A. Hassan H. A. Abdelhafez E.-S. M. N. Abuo-Rahma G. E.-D. A. (2016). Fluoroquinolones as urease inhibitors: anti-*Proteus mirabilis* activity and molecular docking studies. Am. J. Microbiol. Res. 4, 81–84. 10.12691/ajmr-4-3-3

[B5] Abokhalil R. N. Elkhatib W. F. Aboulwafa M. M. Hassouna N. A. (2020). Persisters of *Klebsiella pneumoniae* and *Proteus mirabilis*: a common phenomenon and different behavior Profiles. Curr. Microbiol. 77, 1233–1244. 10.1007/s00284-020-01926-3 32123985

[B6] Ahmed F. Urooj A. (2009). Glucose-lowering, hepatoprotective and hypolipidemic activities of stem bark of Ficus racemosa in streptozotocin-induced diabetic Rats. J. Young Pharm. 1, 160–164. 10.4103/0975-1483.55749

[B7] Aiassa V. Barnes A. Albesa I. (2006). Action of ciprofloxacin on planktonic bacteria and biofilm of *Proteus mirabilis*. Biofilms 3, 11–17. 10.1017/S1479050507002086

[B8] Alav I. Sutton J. M. Rahman K. M. (2018). Role of bacterial efflux pumps in biofilm formation. J. Antimicrob. Chemother. 73, 2003–2020. 10.1093/jac/dky042 29506149

[B9] Allison C. Lai H. C. Hughes C. (1992). Co-ordinate expression of virulence genes during swarm-cell differentiation and population migration of *Proteus mirabilis*. Mol. Microbiol. 6, 1583–1591. 10.1111/j.1365-2958.1992.tb00883.x 1495387

[B10] Alves D. R. Nzakizwanayo J. Dedi C. Olympiou C. Hanin A. Kot W.. (2019). Genomic and ecogenomic characterization of *Proteus mirabilis* bacteriophages. Front. Microbiol. 10:1783. 10.3389/fmicb.2019.01783 31447809 PMC6691071

[B11] Alves D. R. Perez-Esteban P. Kot W. Bean J. E. Arnot T. Hansen L. H.. (2016). A novel bacteriophage cocktail reduces and disperses Pseudomonas aeruginosa biofilms under static and flow conditions. Microb. Biotechnol. 9, 61–74. 10.1111/1751-7915.12316 26347362 PMC4720417

[B12] Aniejurengho O. U. V. (2016). Dendron -Based Synthetic Bacteriophages for the Treatment of Porteus mirabilis Infections. Doctoral Thesis, University of Brighton.

[B13] Armbruster C. E. Mobley H. L. (2012). Merging mythology and morphology: the multifaceted lifestyle of *Proteus mirabilis*. Nat. Rev. Microbiol. 10, 743–754. 10.1038/nrmicro2890 23042564 PMC3621030

[B14] Azeredo J. Sutherland I. W. (2008). The use of phages for the removal of infectious biofilms. Curr. Pharm. Biotechnol. 9, 261–266. 10.2174/138920108785161604 18691087

[B15] Bahrani F. K. Cook S. Hull R. A. Massad G. Mobley H. L. (1993). *Proteus mirabilis* fimbriae: N-terminal amino acid sequence of a major fimbrial subunit and nucleotide sequences of the genes from two strains. Infect. Immun. 61, 884–891. 10.1128/IAI.61.3.884-891.1993 8094384 PMC302815

[B16] Beceiro A. Tomas M. Bou G. (2013). Antimicrobial resistance and virulence: a successful or deleterious association in the bacterial world? Clin. Microbiol. Rev. 26, 185–230. 10.1128/CMR.00059-12 23554414 PMC3623377

[B17] Belas R. Schneider R. Melch M. (1998). Characterization of *Proteus mirabilis* precocious swarming mutants: identification of rsbA, encoding a regulator of swarming behavior. J. Bacteriol. 180, 6126–6139. 10.1128/JB.180.23.6126-6139.1998 9829920 PMC107696

[B18] Belfield K. Bayston R. Birchall J. P. Daniel M. (2015). Do orally administered antibiotics reach concentrations in the middle ear sufficient to eradicate planktonic and biofilm bacteria? A review. Int. J. Pediatr. Otorhinolaryngol. 79, 296–300. 10.1016/j.ijporl.2015.01.003 25623134

[B19] Bentley J. Hyatt L. S. Ainley K. Parish J. H. Herbert R. B. White G. R. (1993). Cloning and sequence analysis of an *Escherichia coli* gene conferring bicyclomycin resistance. Gene 127, 117–120. 10.1016/0378-1119(93)90625-D 8486276

[B20] Bichler K. H. Eipper E. Naber K. Braun V. Zimmermann R. Lahme S. (2002). Urinary infection stones. Int. J. Antimicrob. Agents 19, 488–498. 10.1016/S0924-8579(02)00088-2 12135839

[B21] Brayner R. Ferrari-Iliou R. Brivois N. Djediat S. Benedetti M. F. Fievet F. (2006). Toxicological impact studies based on Escherichia coli bacteria in ultrafine ZnO nanoparticles colloidal medium. Nano Lett. 6, 866–870. 10.1021/nl052326h 16608300

[B22] Brown H. L. Van Vliet A. H. Betts R. P. Reuter M. (2013). Tetrazolium reduction allows assessment of biofilm formation by Campylobacter jejuni in a food matrix model. J. Appl. Microbiol. 115, 1212–1221. 10.1111/jam.12316 23910098

[B23] Burne R. A. Chen Y. Y. (2000). Bacterial ureases in infectious diseases. Microbes. Infect. 2, 533–542. 10.1016/S1286-4579(00)00312-9 10865198

[B24] Buzea C. Pacheco I. I. Robbie K. (2007). Nanomaterials and nanoparticles: Sources and toxicity. Biointerphases 2, MR17–MR71. 10.1116/1.2815690 20419892

[B25] Carson L. Gorman S. P. Gilmore B. F. (2010). The use of lytic bacteriophages in the prevention and eradication of biofilms of *Proteus mirabilis* and Escherichia coli. FEMS Immunol. Med. Microbiol. 59, 447–455. 10.1111/j.1574-695X.2010.00696.x 20528927

[B26] Cerca N. Oliveira R. Azeredo J. (2007). Susceptibility of Staphylococcus epidermidis planktonic cells and biofilms to the lytic action of staphylococcus bacteriophage K. Lett. Appl. Microbiol. 45, 313–317. 10.1111/j.1472-765X.2007.02190.x 17718845

[B27] Chan B. Abedon S. (2014). Bacteriophages and their enzymes in biofilm control. Curr. Pharm. Design 21, 85–99. 10.2174/1381612820666140905112311 25189866

[B28] Chen C. Y. Chen Y. H. Lu P. L. Lin W. R. Chen T. C. Lin C. Y. (2012). *Proteus mirabilis* urinary tract infection and bacteremia: risk factors, clinical presentation, and outcomes. J. Microbiol. Immunol. Infect. 45, 228–236. 10.1016/j.jmii.2011.11.007 22572004

[B29] Chhibber S. Nag D. Bansal S. (2013). Inhibiting biofilm formation by *Klebsiella pneumoniae* B5055 using an iron antagonizing molecule and a bacteriophage. BMC Microbiol. 13:174. 10.1186/1471-2180-13-174 23889975 PMC3726515

[B30] Choo J. H. Rukayadi Y. Hwang J. K. (2006). Inhibition of bacterial quorum sensing by vanilla extract. Lett. Appl. Microbiol. 42, 637–641. 10.1111/j.1472-765X.2006.01928.x 16706905

[B31] Coker C. Poore C. A. Li X. Mobley H. L. (2000). Pathogenesis of *Proteus mirabilis* urinary tract infection. Microbes Infect. 2, 1497–1505. 10.1016/S1286-4579(00)01304-6 11099936

[B32] Cook S. W. Mody N. Valle J. Hull R. (1995). Molecular cloning of *Proteus mirabilis* uroepithelial cell adherence (uca) genes. Infect. Immun. 63, 2082–2086. 10.1128/IAI.63.5.2082-2086.1995 7729924 PMC173269

[B33] De Marco S. Piccioni M. Pagiotti R. Pietrella D. (2017). Antibiofilm and antioxidant activity of propolis and bud poplar resins versus *Pseudomonas aeruginosa*. Evid. Based Complem. Altern. Med. 2017:5163575. 10.1155/2017/5163575 28127379 PMC5239991

[B34] Donlan R. M. (2002). Biofilms: microbial life on surfaces. Emerg. Infect. Dis. 8, 881–890. 10.3201/eid0809.020063 12194761 PMC2732559

[B35] Donlan R. M. (2009). Preventing biofilms of clinically relevant organisms using bacteriophage. Trends Microbiol. 17, 66–72. 10.1016/j.tim.2008.11.002 19162482

[B36] Downer A. Morris N. Feast W. J. Stickler D. (2003). Polymer surface properties and their effect on the adhesion of *Proteus mirabilis*. Proc. Inst. Mech. Eng. H 217, 279–289. 10.1243/095441103322060730 12885198

[B37] Drzewiecka D. (2016). Significance and roles of *Proteus* spp. bacteria in natural environments. Microb. Ecol. 72, 741–758. 10.1007/s00248-015-0720-6 26748500 PMC5080321

[B38] Dumanski A. J. Hedelin H. Edin-Liljegren A. Beauchemin D. Mclean R. J. (1994). Unique ability of the *Proteus mirabilis* capsule to enhance mineral growth in infectious urinary calculi. Infect. Immun. 62, 2998–3003. 10.1128/IAI.62.7.2998-3003.1994 8005688 PMC302911

[B39] Durgadevi R. Veera Ravi A. Alexpandi R. Krishnan Swetha T. Abirami G. Vishnu S.. (2019). Virulence targeted inhibitory effect of linalool against the exclusive uropathogen *Proteus mirabilis*. Biofouling 35, 508–525. 10.1080/08927014.2019.1619704 31144520

[B40] Ermel G. Georgeault S. Inisan C. Besnard M. (2012). Inhibition of adhesion of uropathogenic Escherichia coli bacteria to uroepithelial cells by extracts from cranberry. J. Med. Food. 15, 126–134. 10.1089/jmf.2010.0312 22082066

[B41] Fernandez L. Gonzalez S. Campelo A. B. Martinez B. Rodriguez A. Garcia P. (2017). Low-level predation by lytic phage phiIPLA-RODI promotes biofilm formation and triggers the stringent response in Staphylococcus aureus. Sci Rep 7:40965. 10.1038/srep40965 28102347 PMC5244418

[B42] Flemming H. C. Wingender J. (2010). The biofilm matrix. Nat. Rev. Microbiol. 8, 623–633. 10.1038/nrmicro2415 20676145

[B43] Fraser G. M. Claret L. Furness R. Gupta S. Hughes C. (2002). Swarming-coupled expression of the *Proteus mirabilis* hpmBA haemolysin operon. Microbiology (Reading, England) 148, 2191–2201. 10.1099/00221287-148-7-2191 12101306 PMC2528290

[B44] Freires I. A. Queiroz V. Furletti V. F. Ikegaki M. De Alencar S. M. Duarte M. C. T.. (2016). Chemical composition and antifungal potential of Brazilian propolis against *Candida* spp. J Mycol. Med. 26, 122–132. 10.1016/j.mycmed.2016.01.003 26916845

[B45] Gannabathula S. Skinner M. A. Rosendale D. Greenwood J. M. Mutukumira A. N. Steinhorn G.. (2012). Arabinogalactan proteins contribute to the immunostimulatory properties of New Zealand honeys. Immunopharmacol. Immunotoxicol. 34, 598–607. 10.3109/08923973.2011.641974 22212104

[B46] Gill J. J. Hyman P. (2010). Phage choice, isolation, and preparation for phage therapy. Curr. Pharm. Biotechnol. 11, 2–14. 10.2174/138920110790725311 20214604

[B47] Gorski A. Miedzybrodzki R. Borysowski J. Weber-Dabrowska B. Lobocka M. Fortuna W. (2009). Bacteriophage therapy for the treatment of infections. Curr. Opin. Investig. Drugs. 10, 766–774. 19649921

[B48] Gunalan S. Sivaraj R. Rajendran V. (2012). Green synthesized ZnO nanoparticles against bacterial and fungal pathogens. Progr. Nat. Sci. Mater. Int. 22, 693–700. 10.1016/j.pnsc.2012.11.015

[B49] Guttenplan S. B. Kearns D. B. (2013). Regulation of flagellar motility during biofilm formation. FEMS Microbiol. Rev. 37, 849–871. 10.1111/1574-6976.12018 23480406 PMC3718880

[B50] Hanlon G. W. (2007). Bacteriophages: an appraisal of their role in the treatment of bacterial infections. Int. J. Antimicrob. Agents 30, 118–128. 10.1016/j.ijantimicag.2007.04.006 17566713

[B51] Harper D.G. (2013). Beneficial Effects of Bacteriophage Treatments. United States patent application 8475787.

[B52] Harper D. R. Parracho H. M. R. T. Walker J. Sharp R. Hughes G. Werthén M. (2014). Bacteriophages and biofilms. Antibiotics 3, 270–284. 10.3390/antibiotics3030270

[B53] Harshey R.M. (2003). Bacterial motility on a surface: many ways to a common goal. Annu. Rev. Microbiol. 57, 249–273. 10.1146/annurev.micro.57.030502.091014 14527279

[B54] Hayashi M. Tabata K. Yagasaki M. Yonetani Y. (2010). Effect of multidrug-efflux transporter genes on dipeptide resistance and overproduction in Escherichia coli. FEMS Microbiol. Lett. 304, 12–19. 10.1111/j.1574-6968.2009.01879.x 20067529

[B55] Hayat S. Muzammil S. Rasool M. H. Nisar Z. Hussain S. Z. Sabri A.N.. (2018). *In vitro* antibiofilm and anti-adhesion effects of magnesium oxide nanoparticles against antibiotic resistant bacteria. Microbiol. Immunol. 62, 211–220. 10.1111/1348-0421.12580 29405384

[B56] Hengzhuang W. Wu H. Ciofu O. Song Z. Hoiby N. (2011). Pharmacokinetics/pharmacodynamics of colistin and imipenem on mucoid and nonmucoid *Pseudomonas aeruginosa* biofilms. Antimicrob. Agents Chemother. 55, 4469–4474. 10.1128/AAC.00126-11 21670181 PMC3165294

[B57] Hoiby N. Bjarnsholt T. Givskov M. Molin S. Ciofu O. (2010). Antibiotic resistance of bacterial biofilms. Int. J. Antimicrob. Agents 35, 322–332. 10.1016/j.ijantimicag.2009.12.011 20149602

[B58] Holling N. Lednor D. Tsang S. Bissell A. Campbell L. Nzakizwanayo J.. (2014). Elucidating the genetic basis of crystalline biofilm formation in *Proteus mirabilis*. Infect. Immun. 82, 1616–1626. 10.1128/IAI.01652-13 24470471 PMC3993403

[B59] Horii T. Morita M. Muramatsu H. Muranaka Y. Kanno T. Maekawa M. (2003). Effects of mupirocin at subinhibitory concentrations on flagella formation in *Pseudomonas aeruginosa* and *Proteus mirabilis*. J. Antimicrob. Chemother. 51, 1175–1179. 10.1093/jac/dkg226 12697640

[B60] Iribarnegaray V. Navarro N. Robino L. Zunino P. Morales J. Scavone P. (2019). Magnesium-doped zinc oxide nanoparticles alter biofilm formation of *Proteus mirabilis*. Nanomedicine (Lond) 14, 1551–1564. 10.2217/nnm-2018-0420 31166149

[B61] Irwin N. J. Mccoy C. P. Carson L. (2013). Effect of pH on the *in vitro* susceptibility of planktonic and biofilm-grown *Proteus mirabilis* to the quinolone antimicrobials. J. Appl. Microbiol. 115, 382–389. 10.1111/jam.12241 23647563

[B62] Irzh A. Genish I. Klein L. Solovyov L. A. Gedanken A. (2010). Synthesis of ZnO and Zn nanoparticles in microwave plasma and their deposition on glass slides. Langmuir 26, 5976–5984. 10.1021/la904499s 20337410

[B63] Issac Abraham S. V. Palani A. Ramaswamy B. R. Shunmugiah K. P. Arumugam V. R. (2011). Antiquorum sensing and antibiofilm potential of *Capparis spinosa*. Arch. Med. Res. 42, 658–668. 10.1016/j.arcmed.2011.12.002 22222491

[B64] Jacobsen S. Shirtliff M. (2011). *Proteus mirabilis* biofilms and catheter-associated urinary infections. Virulence 2, 460–465. 10.4161/viru.2.5.17783 21921687

[B65] Jacobsen S. M. Stickler D. J. Mobley H. L. Shirtliff M. E. (2008). Complicated catheter-associated urinary tract infections due to Escherichia coli and *Proteus mirabilis*. Clin. Microbiol. Rev. 21, 26–59. 10.1128/CMR.00019-07 18202436 PMC2223845

[B66] Jamal M. Hussain T. Das C. R. Andleeb S. (2015). Characterization of Siphoviridae phage Z and studying its efficacy against multidrug-resistant *Klebsiella pneumoniae* planktonic cells and biofilm. J. Med. Microbiol. 64, 454–462. 10.1099/jmm.0.000040 25681321

[B67] Jamil R. T. Foris L. A. Snowden J. (2020). Proteus mirabilis infections, in StatPearls. (Treasure Island, FL: StatPearls Publishing). Available online at: https://www.ncbi.nlm.nih.gov/books/NBK442017/28723046

[B68] Jansen A. M. Lockatell V. Johnson D. E. Mobley H. L. (2004). Mannose-resistant Proteus-like fimbriae are produced by most *Proteus mirabilis* strains infecting the urinary tract, dictate the *in vivo* localization of bacteria, and contribute to biofilm formation. Infect. Immun. 72, 7294–7305. 10.1128/IAI.72.12.7294-7305.2004 15557655 PMC529131

[B69] Jenssen H. Hamill P. Hancock R. E. W. (2006). Peptide Antimicrobial Agents. J Clin. Microbiol. Rev. 19, 491–511. 10.1128/CMR.00056-05 PMC1539102 16847082

[B70] Johnson J.R. Johnston B. Kuskowski M.A. (2012). *In vitro* comparison of nitrofurazone- and silver alloy-coated foley catheters for contact-dependent and diffusible inhibition of urinary tract infection-associated microorganisms. Antimicrob. Agents Chemother. 56, 4969–4972. 10.1128/AAC.00733-12 22751541 PMC3421844

[B71] Jonczyk E. Kłak M. Miedzybrodzki R. Górski A. (2011). The influence of external factors on bacteriophages–review. Folia Microbiol. 56, 191–200. 10.1007/s12223-011-0039-8 21625877 PMC3131515

[B72] Jones B.V. Young R. Mahenthiralingam E. Stickler D.J. (2004). Ultrastructure of *Proteus mirabilis* swarmer cell rafts and role of swarming in catheter-associated urinary tract infection. Infect. Immun. 72, 3941–3950. 10.1128/IAI.72.7.3941-3950.2004 15213138 PMC427392

[B73] Jones G. L. Russell A. D. Caliskan Z. Stickler D. J. (2005). A strategy for the control of catheter blockage by crystalline *Proteus mirabilis* biofilm using the antibacterial agent triclosan. Eur. Urol. 48, 838–845. 10.1016/j.eururo.2005.07.004 16126323

[B74] Jones N. Ray B. Ranjit K.T. Manna A.C. (2008). Antibacterial activity of ZnO nanoparticle suspensions on a broad spectrum of microorganisms. FEMS Microbiol. Lett. 279, 71–76. 10.1111/j.1574-6968.2007.01012.x 18081843

[B75] Jones S. M. Yerly J. Hu Y. Ceri H. Martinuzzi R. (2007). Structure of *Proteus mirabilis* biofilms grown in artificial urine and standard laboratory media. FEMS Microbiol. Lett. 268, 16–21. 10.1111/j.1574-6968.2006.00587.x 17250761

[B76] Kaca W. Glenska J. Lechowicz L. Grabowski S. Brauner A. Kwinkowski M. (2011). Serotyping of *Proteus mirabilis* clinical strains based on lipopolysaccharide O-polysaccharide and core oligosaccharide structures. Biochem. Biokhimiia 76, 851–861. 10.1134/S0006297911070169 21999547

[B77] Kregiel D. Rygala A. Kolesinska B. Nowacka M. Herc A. S. Kowalewska A. (2019). Antimicrobial and antibiofilm N-acetyl-L-cysteine grafted siloxane polymers with potential for use in water systems. Int. J. Mol Sci. 20:2011. 10.3390/ijms20082011 31022884 PMC6515369

[B78] Kutter E. De Vos D. Gvasalia G. Alavidze Z. Gogokhia L. Kuhl S.. (2010). Phage therapy in clinical practice: treatment of human infections. Curr. Pharm. Biotechnol. 11, 69–86. 10.2174/138920110790725401 20214609

[B79] Kwiecinska-Pirog J. Bogiel T. Gospodarek E. (2013a). Effects of ceftazidime and ciprofloxacin on biofilm formation in *Proteus mirabilis* rods. J. Antibiot. (Tokyo) 66, 593–597. 10.1038/ja.2013.59 23801185

[B80] Kwiecinska-Pirog J. Bogiel T. Skowron K. Wieckowska E. Gospodarek E. (2014). *Proteus mirabilis* biofilm - qualitative and quantitative colorimetric methods-based evaluation. Braz. J. Microbiol. 45, 1423–1431. 10.1590/S1517-83822014000400037 25763050 PMC4323319

[B81] Kwiecinska-Pirog J. Skowron K. Sniegowska A. Przekwas J. Balcerek M. Zaluski D.. (2019). The impact of ethanol extract of propolis on biofilm forming by *Proteus mirabilis* strains isolated from chronic wounds infections. Nat. Prod. Res. 33, 3293–3297. 10.1080/14786419.2018.1470513 29726711

[B82] Kwiecinska-Pirog J. Skowron K. Zniszczol K. Gospodarek E. (2013b). The assessment of *Proteus mirabilis* susceptibility to ceftazidime and ciprofloxacin and the impact of these antibiotics at subinhibitory concentrations on *Proteus mirabilis* biofilms. Biomed. Res. Int. 2013:930876. 10.1155/2013/930876 24151628 PMC3787586

[B83] Latka A. Maciejewska B. Majkowska-Skrobek G. Briers Y. Drulis-Kawa Z. (2017). Bacteriophage-encoded virion-associated enzymes to overcome the carbohydrate barriers during the infection process. Appl. Microbiol. Biotechnol. 101, 3103–3119. 10.1007/s00253-017-8224-6 28337580 PMC5380687

[B84] Lehman S. M. Donlan R. M. (2015). Bacteriophage-mediated control of a two-species biofilm formed by microorganisms causing catheter-associated urinary tract infections in an *in vitro* urinary catheter model. Antimicrob. Agents Chemother. 59, 1127–1137. 10.1128/AAC.03786-14 25487795 PMC4335898

[B85] Lewis K. (2001). Riddle of biofilm resistance. Antimicrob. Agents Chemother. 45, 999–1007. 10.1128/AAC.45.4.999-1007.2001 11257008 PMC90417

[B86] Lewis K. (2005). Persister cells and the riddle of biofilm survival. Biochemistry (Mosc) 70, 267–274. 10.1007/s10541-005-0111-6 15807669

[B87] Li X. Lu N. Brady H. Packman A. (2016). Ureolytic Biomineralization Reduces *Proteus mirabilis* Biofilm Susceptibility to Ciprofloxacin. Antimicrob. Agents Chemother. 60, AAC.00203–00216. 10.1128/AAC.00203-16 26953206 PMC4862469

[B88] Liaw S. J. Lai H. C. Ho S. W. Luh K. T. Wang W. B. (2003). Role of RsmA in the regulation of swarming motility and virulence factor expression in *Proteus mirabilis*. J. Med. Microbiol. 52, 19–28. 10.1099/jmm.0.05024-0 12488561

[B89] Limoli D. H. Rockel A. B. Host K. M. Jha A. Kopp B. T. Hollis T.. (2014). Cationic antimicrobial peptides promote microbial mutagenesis and pathoadaptation in chronic infections. PLoS Pathog. 10:e1004083. 10.1371/journal.ppat.1004083 24763694 PMC3999168

[B90] Lu Q. Yu J. Yang X. Wang J. Wang L. Lin Y.. (2010). Ambroxol interferes with *Pseudomonas aeruginosa* quorum sensing. Int. J. Antimicrob. Agents 36, 211–215. 10.1016/j.ijantimicag.2010.05.007 20580207

[B91] Majtan J. Bohova J. Horniackova M. Klaudiny J. Majtan V. (2014). Anti-biofilm effects of honey against wound pathogens *Proteus mirabilis* and Enterobacter cloacae. Phytother. Res. 28, 69–75. 10.1002/ptr.4957 23494861

[B92] Malone M. Tsai G. (2016). Wound healing with Apitherapy: a review of the effects of honey. J. Apither. 1:29 10.5455/ja.20160620031837

[B93] Mardirossian M. Grzela R. Giglione C. Meinnel T. Gennaro R. Mergaert P.. (2014). The host antimicrobial peptide Bac71-35 binds to bacterial ribosomal proteins and inhibits protein synthesis. Chem. Biol. 21, 1639–1647. 10.1016/j.chembiol.2014.10.009 25455857

[B94] Massad G. Fulkerson J. F. Jr. Watson D. C. Mobley H. L. (1996). *Proteus mirabilis* ambient-temperature fimbriae: cloning and nucleotide sequence of the aft gene cluster. Infect Immun. 64, 4390–4395. 10.1128/IAI.64.10.4390-4395.1996 8926119 PMC174387

[B95] Maszewska A. Zygmunt M. Grzejdziak I. Rozalski A. (2018). Use of polyvalent bacteriophages to combat biofilm of *Proteus mirabilis* causing catheter-associated urinary tract infections. J. Appl. Microbiol. 125, 1253–1265. 10.1111/jam.14026 29924909

[B96] Mathur S. Sabbuba N.A. Suller M.T. Stickler D.J. Feneley R.C. (2005). Genotyping of urinary and fecal *Proteus mirabilis* isolates from individuals with long-term urinary catheters. Eur. J. Clin. Microbiol. Infect. Dis. 24, 643–644. 10.1007/s10096-005-0003-0 16167137

[B97] Melo L. D. Veiga P. Cerca N. Kropinski A. M. Almeida C. Azeredo J. (2016). Development of a phage cocktail to control *Proteus mirabilis* catheter-associated urinary tract infections. Front. Microbiol. 7:1024 10.3389/fmicb.2016.01024 27446059 PMC4923195

[B98] Messarah M. Klibet F. Boumendjel A. Abdennour C. Bouzerna N. Boulakoud M.S.. (2012). Hepatoprotective role and antioxidant capacity of selenium on arsenic-induced liver injury in rats. Exp. Toxicol. Pathol. 64, 167–174. 10.1016/j.etp.2010.08.002 20851583

[B99] Miedzybrodzki R. Borysowski J. Weber-Dabrowska B. Fortuna W. Letkiewicz S. Szufnarowski K. (2012). Chapter 3 - Clinical aspects of phage therapy, in Advances in Virus Research, eds Łobocka M. Szybalski W. (Cambridge, MA: Academic Press), 73–121. 10.1016/B978-0-12-394438-2.00003-7 22748809

[B100] Miron T. Rabinkov A. Mirelman D. Wilchek M. Weiner L. (2000). The mode of action of allicin: its ready permeability through phospholipid membranes may contribute to its biological activity. Biochim. Biophy. Acta 1463, 20–30. 10.1016/S0005-2736(99)00174-1 10631291

[B101] Morgan S. D. Rigby D. Stickler D.J. (2009). A study of the structure of the crystalline bacterial biofilms that can encrust and block silver Foley catheters. Urol. Res. 37, 89–93. 10.1007/s00240-009-0176-6 19189089

[B102] Morones-Ramirez J. R. Winkler J. A. Spina C. S. Collins J. J. (2013). Silver enhances antibiotic activity against gram-negative bacteria. Sci. Transl. Med. 5:190ra181. 10.1126/scitranslmed.3006276 23785037 PMC3771099

[B103] Morozova V. Kozlova Y. Shedko E. Kurilshikov A. Babkin I. Tupikin A.. (2016). Lytic bacteriophage PM16 specific for *Proteus mirabilis*: a novel member of the genus Phikmvvirus. Arch. Virol. 161, 2457–2472. 10.1007/s00705-016-2944-2 27350061

[B104] Morris N.S. Stickler D.J. Winters C. (1997). Which indwelling urethral catheters resist encrustation by *Proteus mirabilis* biofilms? Br. J. Urol. 80, 58–63. 10.1046/j.1464-410X.1997.00185.x 9240181

[B105] Morris N. S. Stickler D. J. (2001). Does drinking cranberry juice produce urine inhibitory to the development of crystalline, catheter-blocking *Proteus mirabilis* biofilms? BJU Int. 88, 192–197. 10.1046/j.1464-410x.2001.02248.x 11488728

[B106] Nashikkar N. Begde D. Bundale S. Pise M. Rudra J. Upadhyay A. (2011). Inhibition of swarming motility, biofilm formation and virulence factor expression of uropathogens by Euphorbia trigona latex extracts. Int. J. Pharm. Sci. Res. 2, 558–566. 10.13040/IJPSR.0975-8232

[B107] Nicolle L.E. (2014). Catheter associated urinary tract infections. Antimicrob. Resist. Infect. Control. 3:23. 10.1186/2047-2994-3-23 25075308 PMC4114799

[B108] Nicolosi D. Tempera G. Genovese C. Furneri P. M. (2014). Anti-adhesion activity of A2-type proanthocyanidins (a cranberry major component) on uropathogenic *E. coli* and *P. mirabilis* strains. Antibiotics (Basel) 3, 143–154. 10.3390/antibiotics3020143 27025740 PMC4790394

[B109] Nowack R. Schmitt W. (2008). Cranberry juice for prophylaxis of urinary tract infections–conclusions from clinical experience and research. Phytomedicine 15, 653–667. 10.1016/j.phymed.2008.07.009 18691859

[B110] Nzakizwanayo J. Hanin A. Alves D. R. Mccutcheon B. Dedi C. Salvage J.. (2015). Bacteriophage can prevent encrustation and blockage of urinary catheters by *Proteus mirabilis*. Antimicrob. Agents Chemother. 60, 1530–1536. 10.1128/AAC.02685-15 26711744 PMC4775969

[B111] Nzakizwanayo J. Scavone P. Jamshidi S. Hawthorne J. A. Pelling H. Dedi C.. (2017). Fluoxetine and thioridazine inhibit efflux and attenuate crystalline biofilm formation by *Proteus mirabilis*. Sci. Rep. 7:12222. 10.1038/s41598-017-12445-w 28939900 PMC5610337

[B112] O'hara C. M. Brenner F. W. Miller J. M. (2000). Classification, identification, and clinical significance of Proteus, Providencia, and Morganella. Clin. Microbiol. Rev. 13, 534–546. 10.1128/CMR.13.4.534 11023955 PMC88947

[B113] Old D. C. Adegbola R. A. (1982). Haemagglutinins and fimbriae of Morganella, Proteus and Providencia. J. Med. Microbiol. 15, 551–564. 10.1099/00222615-15-4-551 6129324

[B114] O'may C. Amzallag O. Bechir K. Tufenkji N. (2016). Cranberry derivatives enhance biofilm formation and transiently impair swarming motility of the uropathogen *Proteus mirabilis* HI4320. Can. J. Microbiol. 62, 464–474. 10.1139/cjm-2015-0715 27090825

[B115] O'toole G. Kaplan H.B. Kolter R. (2000). Biofilm formation as microbial development. Annu. Rev. Microbiol. 54, 49–79. 10.1146/annurev.micro.54.1.49 11018124

[B116] Packiavathy I.A. Priya S. Pandian S.K. Ravi A.V. (2014). Inhibition of biofilm development of uropathogens by curcumin - an anti-quorum sensing agent from Curcuma longa. Food Chem. 148, 453–460. 10.1016/j.foodchem.2012.08.002 24262582

[B117] Peana A. T. D'aquila P. S. Panin F. Serra G. Pippia P. Moretti M. D. (2002). Anti-inflammatory activity of linalool and linalyl acetate constituents of essential oils. Phytomedicine 9, 721–726. 10.1078/094471102321621322 12587692

[B118] Pearson M. M. Sebaihia M. Churcher C. Quail M. A. Seshasayee A. S. Luscombe N. M.. (2008). Complete genome sequence of uropathogenic *Proteus mirabilis*, a master of both adherence and motility. J. Bacteriol. 190, 4027–4037. 10.1128/JB.01981-07 18375554 PMC2395036

[B119] Penner J. L. (2005). Genus XXIX. Proteus, in Bergey's Manual of Systematic Bacteriology. The Proteobacteria: Part B, the Gammaproteobacteria, 2nd Edn., eds Brenner D. J. Krieg N. R. Staley J. T. Garrity G. M. (Philadelphia, PA: Lippincott Williams & Wilkins), 745–753.

[B120] Pereira C. S. Thompson J. A. Xavier K. B. (2013). AI-2-mediated signalling in bacteria. FEMS Microbiol. Rev. 37, 156–181. 10.1111/j.1574-6976.2012.00345.x 22712853

[B121] Pradeev Raj K. Sadaiyandi K. Kennedy A. Sagadevan S. Chowdhury Z. Z. Johan M. R. B.. (2018). Influence of Mg doping on ZnO nanoparticles for enhanced photocatalytic evaluation and antibacterial analysis. Nanoscale Res. Lett. 13:229. 10.1186/s11671-018-2643-x 30076473 PMC6081874

[B122] Pushpakom S. Iorio F. Eyers P. A. Escott K. J. Hopper S. Wells A.. (2019). Drug repurposing: progress, challenges and recommendations. Nat. Rev. Drug. Discov. 18, 41–58. 10.1038/nrd.2018.168 30310233

[B123] Rajivgandhi G. Vijayan R. Maruthupandy M. Vaseeharan B. Manoharan N. (2018). Antibiofilm effect of *Nocardiopsis* sp. GRG 1 (KT235640) compound against biofilm forming Gram negative bacteria on UTIs. Microb. Pathog. 118, 190–198. 10.1016/j.micpath.2018.03.011 29524549

[B124] Ranjbar-Omid M. Arzanlou M. Amani M. Shokri Al-Hashem S. K. Amir Mozafari N. Peeri Doghaheh H. (2015). Allicin from garlic inhibits the biofilm formation and urease activity of *Proteus mirabilis in vitro*. FEMS Microbiol. Lett. 362:fnv049. 10.1093/femsle/fnv049 25837813

[B125] Roberts J.A. Fussell E.N. Kaack M.B. (1990). Bacterial adherence to urethral catheters. J. Urol. 144, 264–269. 10.1016/S0022-5347(17)39428-4 2115595

[B126] Robertson M. H. Norton M. S. (1990). Effect of 1% mandelic acid as a bladder irrigation fluid in patients with in-dwelling catheters. Br. J. Clin. Pract. 44, 142–144. 2196927

[B127] Rocha S. P. Pelayo J. S. Elias W. P. (2007). Fimbriae of uropathogenic *Proteus mirabilis*. FEMS Immunol. Med. Microbiol. 51, 1–7. 10.1111/j.1574-695X.2007.00284.x 17640292

[B128] Rosen R. T. Hiserodt R. D. Fukuda E. K. Ruiz R. J. Zhou Z. Lech J.. (2001). Determination of allicin, S-allylcysteine and volatile metabolites of garlic in breath, plasma or simulated gastric fluids. J. Nutr. 131, 968S−971S. 10.1093/jn/131.3.968S 11238798

[B129] Rozalski A. Sidorczyk Z. Kotelko K. (1997). Potential virulence factors of Proteus bacilli. Microbiol. Mol. Biol. Rev. 61, 65–89. 10.1128/.61.1.65-89.1997 9106365 PMC232601

[B130] Sabaeifard P. Abdi-Ali A. Soudi M.R. Dinarvand R. (2014). Optimization of tetrazolium salt assay for *Pseudomonas aeruginosa* biofilm using microtiter plate method. J. Microbiol. Methods 105, 134–140. 10.1016/j.mimet.2014.07.024 25086178

[B131] Sakuraoka R. Suzuki T. Morohoshi T. (2019). Distribution and genetic diversity of genes involved in quorum sensing and prodigiosin biosynthesis in the complete genome sequences of Serratia marcescens. Genome Biol. Evol. 11, 931–936. 10.1093/gbe/evz046 30840067 PMC6433178

[B132] Salini R. Sindhulakshmi M. Poongothai T. Pandian S.K. (2015). Inhibition of quorum sensing mediated biofilm development and virulence in uropathogens by Hyptis suaveolens. Antonie Van Leeuwenhoek 107, 1095–1106. 10.1007/s10482-015-0402-x 25656290

[B133] Sareneva T. Holthofer H. Korhonen T.K. (1990). Tissue-binding affinity of *Proteus mirabilis* fimbriae in the human urinary tract. Infect. Immun. 58, 3330–3336. 10.1128/IAI.58.10.3330-3336.1990 1976113 PMC313658

[B134] Savolainen K. Pylkkänen L. Norppa H. Falck G. Lindberg H. Tuomi T. (2010). Nanotechnologies, engineered nanomaterials and occupational health and safety – a review. Safety Sci. 48, 957–963. 10.1016/j.ssci.2010.03.006

[B135] Sawai J. (2003). Quantitative evaluation of antibacterial activities of metallic oxide powders (ZnO, MgO and CaO) by conductimetric assay. J. Microbiol. Methods 54, 177–182. 10.1016/S0167-7012(03)00037-X 12782373

[B136] Scavone P. Iribarnegaray V. Caetano A.L. Schlapp G. Hartel S. Zunino P. (2016). Fimbriae have distinguishable roles in *Proteus mirabilis* biofilm formation. Pathog. Dis. 74:ftw033. 10.1093/femspd/ftw033 27091004

[B137] Schaffer J. N. Pearson M.M. (2015). *Proteus mirabilis* and urinary tract infections. Microbiol. Spectr. 3, 1–66. 10.1128/microbiolspec.UTI-0017-2013 26542036 PMC4638163

[B138] Schneider R. Lockatell C. Johnson D. Belas R. (2002). Detection and mutation of a luxS-encoded autoinducer in *Proteus mirabilis*. Microbiology (Reading, England) 148, 773–782. 10.1099/00221287-148-3-773 11882712

[B139] Schumm K. Lam T.B. (2008). Types of urethral catheters for management of short-term voiding problems in hospitalized adults: a short version Cochrane review. Neurourol. Urodyn. 27, 738–746. 10.1002/nau.20645 18951451

[B140] Sethupathy S. Ananthi S. Selvaraj A. Shanmuganathan B. Vigneshwari L. Balamurugan K.. (2017). Vanillic acid from Actinidia deliciosa impedes virulence in Serratia marcescens by affecting S-layer, flagellin and fatty acid biosynthesis proteins. Sci. Rep. 7:16328. 10.1038/s41598-017-16507-x 29180790 PMC5703977

[B141] Shakibaie M. Forootanfar H. Golkari Y. Mohammadi-Khorsand T. Shakibaie M.R. (2015). Anti-biofilm activity of biogenic selenium nanoparticles and selenium dioxide against clinical isolates of Staphylococcus aureus, *Pseudomonas aeruginosa*, and *Proteus mirabilis*. J. Trace Elem. Med. Biol. 29, 235–241. 10.1016/j.jtemb.2014.07.020 25175509

[B142] Shakibaie M. Khorramizadeh M. R. Faramarzi M. A. Sabzevari O. Shahverdi A. R. (2010). Biosynthesis and recovery of selenium nanoparticles and the effects on matrix metalloproteinase-2 expression. Biotechnol. Appl. Biochem. 56, 7–15. 10.1042/BA20100042 20408816

[B143] Shi X. Wang S. Sun H. Baker J. R. (2007). Improved biocompatibility of surface functionalized dendrimer-entrapped gold nanoparticles. Soft Matter 3, 71–74. 10.1039/B612972B 32680194

[B144] Sillankorva S. Neubauer P. Azaredo J. (2011). Use of Bacteriophages to Control Biofilms. Saarbrücken: LAP Lambert Academic Publishing.

[B145] Sillankorva S. Oliveira R. Vieira M.J. Sutherland I. Azeredo J. (2004). Pseudomonas fluorescens infection by bacteriophage PhiS1: the influence of temperature, host growth phase and media. FEMS Microbiol. Lett. 241, 13–20. 10.1016/j.femsle.2004.06.058 15556704

[B146] Singha P. Locklin J. Handa H. (2016). A review of the recent advances in antimicrobial coatings for urinary catheters. Acta Biomater. 50, 20–40. 10.1016/j.actbio.2016.11.070 27916738 PMC5316300

[B147] Skurnik M. Pajunen M. Kiljunen S. (2007). Biotechnological challenges of phage therapy. Biotechnol. Lett. 29, 995–1003. 10.1007/s10529-007-9346-1 17364214

[B148] Sosa V. Zunino P. (2009). Effect of Ibicella lutea on uropathogenic *Proteus mirabilis* growth, virulence, and biofilm formation. J. Infect. Dev. Ctries 3, 762–770. 10.3855/jidc.232 20009277

[B149] Stafford N. (2011). Switzerland is to fund complementary therapies for six years while effectiveness is evaluated. BMJ 342:d819. 10.1136/bmj.d819 21300707

[B150] Stankowska D. Czerwonka G. Rozalska S. Grosicka M. Dziadek J. Kaca W. (2012). Influence of quorum sensing signal molecules on biofilm formation in *Proteus mirabilis* O18. Folia Microbiol. (Praha) 57, 53–60. 10.1007/s12223-011-0091-4 22198843 PMC3297748

[B151] Stensballe J. Tvede M. Looms D. Lippert F. K. Dahl B. Tonnesen E.. (2007). Infection risk with nitrofurazone-impregnated urinary catheters in trauma patients: a randomized trial. Ann. Intern. Med. 147, 285–293. 10.7326/0003-4819-147-5-200709040-00002 17785483

[B152] Stickler D. Hewett P. (1991). Activity of antiseptics against biofilms of mixed bacterial species growing on silicone surfaces. Eur. J. Clin. Microbiol. Infect. Dis. 10, 157–162. 10.1007/BF01964448 1905626

[B153] Stickler D. Hughes G. (1999). Ability of *Proteus mirabilis* to swarm over urethral catheters. Eur. J. Clin. Microbiol. Infect. Dis. 18, 206–208. 10.1007/s100960050260 10357056

[B154] Stickler D. J. Jones G. L. Russell A. D. (2003). Control of encrustation and blockage of Foley catheters. Lancet 361, 1435–1437. 10.1016/S0140-6736(03)13104-2 12727400

[B155] Tempera G. Corsello S. Genovese C. Caruso F. E. Nicolosi D. (2010). Inhibitory activity of cranberry extract on the bacterial adhesiveness in the urine of women: an *ex-vivo* study. Int. J. Immunopathol. Pharmacol. 23, 611–618. 10.1177/039463201002300223 20646356

[B156] Thompson R.W. (2018). The Isolation and Characterisation of Proteus Mirabilis Bacteriophages and their Effect on the Colonisation and Blockage of Urinary CatheterS. Doctor of Philosophy, University of the West of England.

[B157] Tolson D. L. Barrigar D. L. Mclean R. J. Altman E. (1995). Expression of a nonagglutinating fimbria by *Proteus mirabilis*. Infect. Immun. 63, 1127–1129. 10.1128/IAI.63.3.1127-1129.1995 7868237 PMC173121

[B158] Tseng B. S. Zhang W. Harrison J. J. Quach T. P. Song J. L. Penterman J.. (2013). The extracellular matrix protects *Pseudomonas aeruginosa* biofilms by limiting the penetration of tobramycin. Environ. Microbiol. 15, 2865–2878. 10.1111/1462-2920.12155 23751003 PMC4045617

[B159] Vaidyanathan S. Soni B. M. Hughes P. L. Singh G. Oo T. (2010). Severe ventral erosion of penis caused by indwelling urethral catheter and inflation of Foley balloon in urethra-need to create list of “never events in spinal cord injury” in order to prevent these complications from happening in paraplegic and tetraplegic patients. Adv. Urol. 2010:461539. 10.1155/2010/461539 20671998 PMC2905713

[B160] Verma V. Harjai K. Chhibber S. (2009). Characterization of a T7-like lytic bacteriophage of *Klebsiella pneumoniae* B5055: a potential therapeutic agent. Curr. Microbiol. 59, 274–281. 10.1007/s00284-009-9430-y 19484297

[B161] Viertel T. M. Ritter K. Horz H. P. (2014). Viruses versus bacteria-novel approaches to phage therapy as a tool against multidrug-resistant pathogens. J. Antimicrob. Chemother. 69, 2326–2336. 10.1093/jac/dku173 24872344

[B162] Wasfi R. Abd El-Rahman O. A. Mansour L. E. Hanora A. S. Hashem A. M. Ashour M. S. (2012). Antimicrobial activities against biofilm formed by *Proteus mirabilis* isolates from wound and urinary tract infections. Indian J. Med. Microbiol. 30, 76–80. 10.4103/0255-0857.93044 22361765

[B163] Weidenmaier C. Peschel A. (2008). Teichoic acids and related cell-wall glycopolymers in Gram-positive physiology and host interactions. Nat. Rev. Microbiol. 6, 276–287. 10.1038/nrmicro1861 18327271

[B164] Williams G. J. Stickler D. J. (2008). Effect of triclosan on the formation of crystalline biofilms by mixed communities of urinary tract pathogens on urinary catheters. J. Med. Microbiol. 57, 1135–1140. 10.1099/jmm.0.2008/002295-0 18719184

[B165] Wray S. K. Hull S. I. Cook R. G. Barrish J. Hull R. A. (1986). Identification and characterization of a uroepithelial cell adhesin from a uropathogenic isolate of *Proteus mirabilis*. Infect. Immun. 54, 43–49. 10.1128/IAI.54.1.43-49.1986 2875952 PMC260114

[B166] Wu H. Moser C. Wang H.-Z. Høiby N. Song Z.-J. (2015). Strategies for combating bacterial biofilm infections. Int. J. Oral Sci. 7, 1–7. 10.1038/ijos.2014.65 25504208 PMC4817533

[B167] Wurpel D. J. Totsika M. Allsopp L. P. Webb R. I. Moriel D. G. Schembri M. A. (2016). Comparative proteomics of uropathogenic *Escherichia coli* during growth in human urine identify UCA-like (UCL) fimbriae as an adherence factor involved in biofilm formation and binding to uroepithelial cells. J. Proteomics 131, 177–189. 10.1016/j.jprot.2015.11.001 26546558

[B168] Xu Y. Wang J. Hao Z. Wang S. Liang C. (2019). Biodegradable ciprofloxacin-incorporated waterborne polyurethane polymers prevent bacterial biofilm formation *in vitro*. Exp. Therap. Med. 17, 1831–1836. 10.3892/etm.2018.7113 30783456 PMC6364193

[B169] Yatsunenko T. Rey F. E. Manary M. J. Trehan I. Dominguez-Bello M. G. Contreras M.. (2012). Human gut microbiome viewed across age and geography. Nature 486, 222–227. 10.1038/nature11053 22699611 PMC3376388

[B170] Yazdi M. Bouzari M. Ghaemi E.A. (2018). Isolation and characterization of a lytic bacteriophage (vB_PmiS-TH) and its application in combination with ampicillin against planktonic and biofilm forms of *Proteus mirabilis* isolated from urinary tract infection. J. Mol. Microbiol. Biotechnol. 28, 37–46. 10.1159/000487137 29617701

[B171] Zhai X. Zhang C. Zhao G. Stoll S. Ren F. Leng X. (2017). Antioxidant capacities of the selenium nanoparticles stabilized by chitosan. J. Nanobiotechnol. 15:4. 10.1186/s12951-016-0243-4 28056992 PMC5217424

[B172] Zunino P. Geymonat L. Allen A.G. Legnani-Fajardo C. Maskell D.J. (2000). Virulence of a *Proteus mirabilis* ATF isogenic mutant is not impaired in a mouse model of ascending urinary tract infection. FEMS Immunol. Med. Microbiol. 29, 137–143. 10.1111/j.1574-695X.2000.tb01516.x 11024353

